# How to Avoid Injury to the Renal Artery due to Inclusion of Superior Mesenteric Artery Patch during Organ Procurement

**Published:** 2017-08-01

**Authors:** B. V. Dasari, S. Asthana, N. Ahmad

**Affiliations:** Division of Surgery, Department of Transplantation, St James’s University Hospital, Leeds, United Kingdom

Data from UK National Transplant database indicates that 0.8%–1% of the kidneys are discarded due to organ damage with a 7% reported incidence of injuries during recovery of kidneys, of which 4.8% were renal vascular injuries related to procurement [1]. Whilst vascular injuries (arterial and venous) could be dealt with various reconstructions and modified anastomotic techniques [2], these could potentially lead to complications such as stenosis and rarely, thrombosis. Renal artery stenosis (RAS) of the anastomosis may lead to deterioration of graft function, hypertension and graft failure. 

In deceased donor kidney procurement, renal arteries are procured with a segment of aorta around the renal artery orifice (Carrel’s Patch [CP]). The presence of a CP facilitates renal artery anastomosis in the recipient and helps to minimize anastomotic RAS. It is a common practice in liver and pancreas procurement for the superior mesenteric artery (SMA) to be recovered with a CP. This often leads to a deficient CP on the anterosuperior aspect of the renal arteries and at worst, damage to the main renal artery (Figs 1a and 1b). The left renal artery is more often affected due to its anatomic disposition.

**Figure 1:**
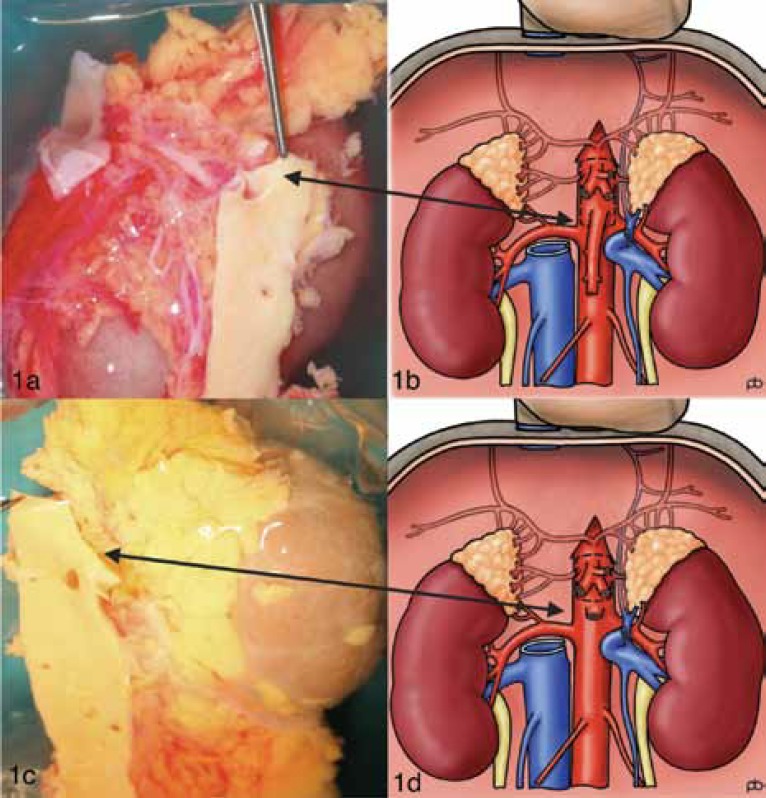
Procurement of the superior mesenteric artery (SMA) with the Carrel’s patch in abdominal multiorgan procurement: Panels 1a and 1b show potential damage to the renal artery patch; panels 1c and 1d depict safe procurement of the renal arteries when the SMA is procured without the Carrel’s patch. Illustrations 1b & 1d, courtesy of Paul Brown.

In a questionnaire-based survey of the liver transplant surgeons in the UK and Ireland, respectively, 20 of 33 surgeons responded to the questionnaire (response rate of 64%). Majority (67%) of respondents were of the opinion that SMA does not require to be procured with liver except when there is an ARHA or a RRHA originating from the SMA. A small proportion (33%) felt that the SMA should always be procured with the liver. When the SMA was procured, 81% of surgeons believed that taking a CP was not required. None of the surgeons used the CP even when recovered in their routine practice of liver transplantation.

Currently over 70% of organ procurement in the UK is performed by senior surgical trainees in transplantation and hepatobiliary surgery, the remaining 30% being performed by the consultants [3]. Recovery of pancreas needs SMA along with it. Generally, liver does not require procurement of SMA except in about 12% of cases where an ARHA or a RRHA originates from the SMA [4]. Often, pancreas will not be recovered in these cases or will be recovered with meticulous division of SMA to preserve both ARHA and superior pancreaticoduodenal artery. In either case, the procurement of SMA should not require a CP. A CP is almost never used in liver or pancreas transplantation. Conversely, a CP facilitates safe anastomosis of the renal artery in renal transplantation, therefore reducing the risk of RAS. When renal artery is used without a CP, a higher incidence of anastomotic RAS has been reported [5-7]. We recommend that during multiorgan abdominal procurement, when the SMA is procured, it should be divided almost flush to the aorta, therefore preserving the aortic patch to be procured with the renal arteries (Figs 1c and 1d).

## References

[1] Ausania F, White SA, Pocock P (2012). Kidney damage during organ recovery in donation after circulatory death donors: data from UK National Transplant Database. Am J Transplant.

[2] Pfeiffer T, Sandman W, Luther B (2002). Vascular surgery for recipient preparation, improved graft quality and acceptability and therapy of ischaemic graft damage in kidney transplantation. Transplantation Proceedings.

[3] National organ retrieval service NORS review update report http://www.nhsbt.nhs.uk/download/board_papers/july14/m14_76_National_Organ_Retrieval_Service_NORS_Review_Update_Report.pdf.

[4] Cherian PT, Hegab B, Oliff SP (2010). The management of an accessory or replaced right hepatic artery during multiorgan retrieval: results of an angiographic study. Liver Transplantation.

[5] Rengel M, Gomes-Da-Silva G, Incháustegui L (1998). Renal artery stenosis after kidney transplantation: diagnostic and therapeutic approach. Kidney Int.

[6] Fung LC, McLorie GA, Khoury AE, Churchill BM (1995). Donor aortic cuff reduces the rate of anastomotic arterial stenosis in pediatric renal transplantation. J Urol.

[7] Belzer F, Glass N, Sollinger H, Morris P (1988). Technical complications after renal transplantation.

